# Evidence-based follow-up in axial spondyloarthritis: how much is (not) enough?

**DOI:** 10.1097/BOR.0000000000001158

**Published:** 2026-03-16

**Authors:** Marius L. Smits, Casper Webers, Astrid van Tubergen

**Affiliations:** a Department of Rheumatology, Maastricht University Medical Centre+; b Care and Public Health Research Institute (CAPHRI); c Department of Epidemiology, Maastricht University, Maastricht, The Netherlands

**Keywords:** axial spondyloarthritis, patient-initiated care, remote monitoring

## Abstract

**Purpose of review:**

Present follow-up strategies for axial spondyloarthritis (axSpA) entail frequent preplanned in-person outpatient visits. Capacity constraints make this approach increasingly difficult, and it is plausible that regular follow-up in axSpA is not necessary for all patients. The purpose of this review is to discuss emerging alternative follow-up strategies to improve care efficiency in axSpA, and the challenges of applying these solutions in clinical practice.

**Recent findings:**

Patient-initiated follow-up (PIFU) and remote monitoring have been investigated in axSpA in two recent trials. These strategies demonstrated meaningful reductions in the number of outpatient visits and associated healthcare costs in patients with stable (ax)SpA, without negatively affecting health outcomes. Qualitative studies have additionally shown widespread acceptability of PIFU and remote monitoring from the perspective of both patients and healthcare providers, and their ability to enhance self-efficacy of patients. Loss to follow-up, delayed care, and logistical limitations are associated concerns requiring further consideration.

**Summary:**

PIFU and remote monitoring hold potential as effective, efficient, and safe strategies for the follow-up of patients with axSpA. For successful implementation in practice, their inherent challenges must be addressed, including the careful selection and training of patients, and ensuring the necessary infrastructure.

## INTRODUCTION

The chronic nature of axial spondyloarthritis (axSpA) often requires a lifelong approach to follow-up. As such, recommendations for disease management highlight the importance of regular monitoring, with the goal of attaining and retaining an inactive disease (ID), or at minimum, low disease activity (LDA) state [1,2]. Currently, this entails frequent preplanned in-person follow-up at the outpatient clinic, through the application of patient-reported outcomes (PROs), clinical findings, laboratory tests, and imaging [1].

However, many clinics face capacity constraints in providing care for patients with axSpA and other rheumatic and musculoskeletal disorders due to rising healthcare expenditures, shortage of rheumatologists, and a gap between workforce supply and demand, that is expected to widen in coming years [3,4]. It is plausible that not all patients with axSpA require regular preplanned follow-up. Scheduled visits for a condition like axSpA, with an unpredictable disease course, are unlikely to coincide with disease flare-ups, raising questions about the usefulness of this follow-up strategy. Alternative approaches may be feasible, particularly in patients with stable disease. In this review, we discuss an evidence-based appraisal of emerging solutions and the key challenges of applying these solutions in the follow-up of patients with axSpA. 

**Box 1:**
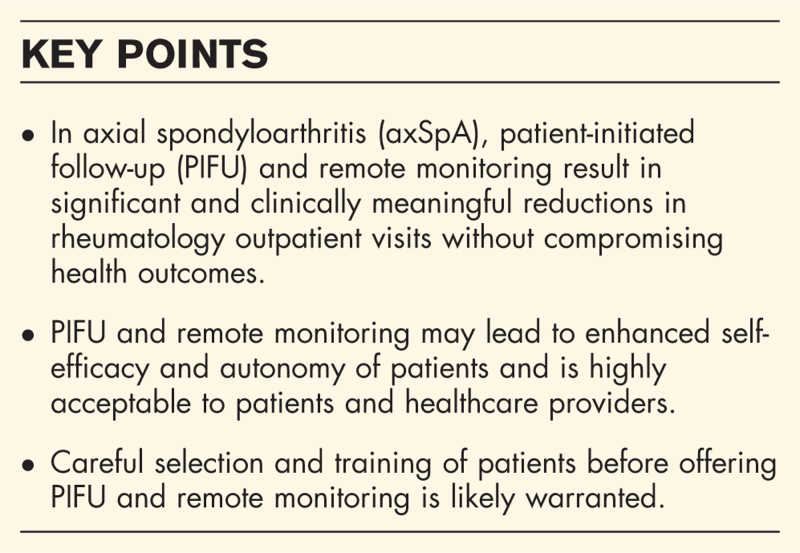
no caption available

## CURRENT SITUATION AND EMERGING SOLUTIONS

The consequences of capacity constraints in rheumatology care are manifold. Prominently, this has led to extensive waiting lists for new patients and reduced ability to provide rapid access to patients during flares or when experiencing side effects, which may compromise quality of care [5–8]. Additional efforts, such as improving care efficiency, are therefore needed to ensure timely patient access to the clinic and manage the workloads of healthcare providers (HCPs).

The traditional follow-up of patients with axSpA is typically physician-initiated, with outpatient visits scheduled every 3–6 months. However, this strategy represents a suboptimal use of time and resources. An observational study in clinical practice showed that approximately one-third of routine outpatient visits of patients with SpA were considered unnecessary in retrospect by rheumatologists [9]. During these visits, antirheumatic therapy remained unchanged in 87% of cases, and 77% of these visits did not lead to any clinical actions. Visits were more likely to be considered unnecessary when disease activity was low and patient satisfaction [based on patient global assessment of disease activity (PtGA)] was high.

Alternative care models, such as patient-initiated follow-up (PIFU) and remote monitoring, may provide solutions to the inefficiencies of traditional follow-up strategies. PIFU enables patients to schedule appointments according to their needs, rather than following a fixed schedule. This approach may empower patients, avoid unnecessary visits, and offer capacity for new and urgent appointments [10]. However, it also carries risks in loss to follow-up and delayed care [11]. Therefore, digital solutions, including telemedicine (TM) and mobile health applications to remotely monitor patients, are increasingly being introduced to support PIFU, either in a synchronous form (involving real-time virtual interactions between patients and HCPs) or asynchronous form (without real-time interactions).

## EVIDENCE FOR PATIENT-INITIATED FOLLOW-UP AND REMOTE MONITORING IN AXIAL SPONDYLOARTHRITIS

**FIGURE 1:**
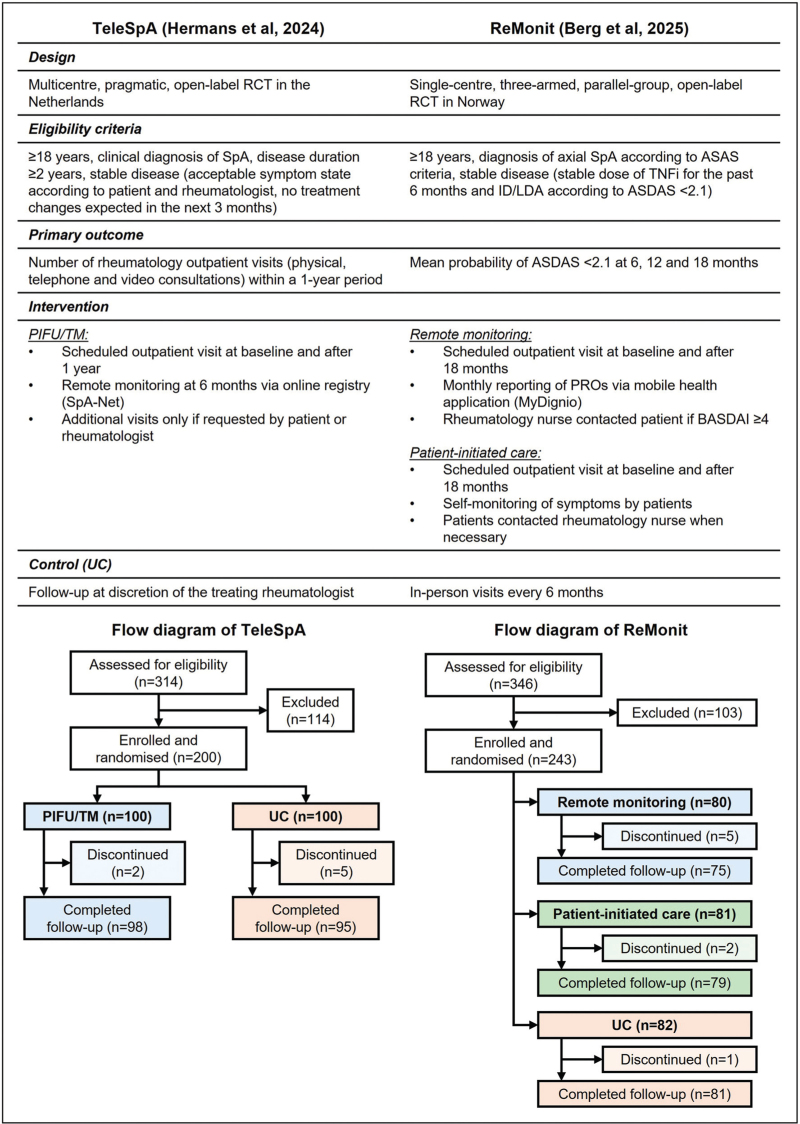
Overview of the TeleSpA and Remote Monitoring in Axial Spondyloarthritis (ReMonit) studies. ASAS, Assessment of SpondyloArthritis international Society; ASDAS, Axial Spondyloarthritis Disease Activity Score; BASDAI, Bath Ankylosing Spondylitis Disease Activity Index; ID, inactive disease; LDA, low disease activity; PIFU, patient-initiated follow-up; PRO, patient-reported outcome; RCT, randomized controlled trial; SpA, spondyloarthritis; TM, telemedicine; TNFi, tumour necrosis factor inhibitor; UC, usual care.

PIFU and remote monitoring have recently been studied in (ax)SpA in two randomized controlled trials (RCTs) (Figure 1). The first, the TeleSpA trial, was a multicentre, pragmatic, open-label RCT, which investigated the clinical and cost-effectiveness of PIFU supported by asynchronous telemedicine (PIFU/TM) for patients with SpA compared to usual care (UC) [12^▪▪^,^13^]. Patients with axSpA (36%), peripheral SpA (54%, including psoriatic arthritis), or combined axial and peripheral SpA (10%) were eligible if they had stable disease (acceptable symptom state according to patient and treating rheumatologist, with no treatment changes expected in the next 3 months). Patients were randomly assigned to either the PIFU/TM or UC group. All patients visited the outpatient clinic at baseline and after 1 year. In the PIFU/TM group, no additional preplanned visits were scheduled, with remote monitoring via TM at 6 months instead. In the UC group, additional visits in-between were scheduled at the discretion of the treating rheumatologist. Extra visits could be scheduled on-demand at any time by patients in both groups. The primary outcome was the between-group difference in number of rheumatology outpatient visits (in-person, telephone, and video) after 1 year. Secondary outcomes were noninferiority of PIFU/TM compared with UC regarding several health outcomes of interest (related to disease activity) and overall experience with care, as well as cost-effectiveness of PIFU/TM. Adverse events were recorded and assessed on seriousness and relatedness to the intervention.

For the primary outcome, the mean number of rheumatology visits was 1.9 (SD 1.5) in the PIFU/TM group and 2.6 (SD 1.3) in the UC group after 1 year (mean difference −0.7 [95% CI −1.0 to −0.3]; 25.4% reduction). At the 6-month TM assessment, 74 of 94 (79%) patients from the PIFU/TM group indicated that no visit or contact was needed, 16 (17%) requested a telephone call, and 4 (4%) an outpatient visit. Regarding secondary outcomes, noninferiority was shown for all outcomes of interest. PIFU/TM was cost-effective from a healthcare perspective, saving costs (−€180 [95% CI −921 to 560]) without a loss in quality-adjusted life years (+0.004 [95% CI -0.022 to 0.030]). Seven nontrial-related adverse events occurred in the PIFU/TM group and eight (including one death) occurred in the UC group.

The second RCT, the Remote Monitoring in Axial Spondyloarthritis (ReMonit) trial, was a single-centre, three-armed, parallel-group, open-label RCT, comparing remote monitoring (comparable to asynchronous TM), PIFU, and UC [14^▪▪^]. Patients with axSpA were eligible if they were in an LDA state [Axial Spondyloarthritis Disease Activity Score (ASDAS) < 2.1] at time of inclusion and had been on stable treatment with a TNF inhibitor for ≥6 months. All participants were assessed at baseline and after 18 months. In the UC group, follow-up visits with a rheumatology nurse (supervised by a rheumatologist) were planned every 6 months in-between. In the remote monitoring and PIFU groups, no preplanned visits were scheduled during follow-up. Instead, in the remote monitoring group, participants completed PROs every month [PtGA, flare question, and Bath Ankylosing Spondylitis Disease Activity Index (BASDAI) if PtGA was ≥ 3 or a flare was suspected]. A BASDAI ≥ 4 alerted the rheumatology nurse to contact the patient and assess the need for a visit. In the PIFU group, patients self-monitored their symptoms and could contact the rheumatology nurse when necessary (e.g. symptom worsening or adverse event). The primary outcome was noninferiority of remote monitoring or PIFU compared to UC for maintaining disease control (ASDAS < 2.1; 15% noninferiority margin) at 6, 12, and 18 months. Secondary outcomes included disease activity, pain, physical function, and satisfaction with care, among others. Number of visits (in-person and telephone), HCP contacts, and adverse events were also recorded.

In all three groups, ≥90% of patients were in an LDA state at 6, 12, and 18 months. For the primary outcome, noninferiority for disease control was demonstrated in both intervention groups, with between-group differences in probability of maintaining LDA of −4.1% (97.5% CI −9.9% to 1.8%) for UC versus remote monitoring, and −1.1% (97.5% CI −7.2% to 4.9%) for UC versus PIFU. Furthermore, PIFU was noninferior to remote monitoring regarding disease control. Secondary outcomes were also similar between groups. Both remote monitoring and PIFU led to a reduction in the total number of visits compared to UC, and the number of unscheduled visits in the three groups was comparable. Of note, when brief telephone contacts were also considered, these were substantially higher in the remote monitoring group (by design of this intervention, where the nurse contacted the patient if BASDAI ≥ 4).

Overall, these two trials demonstrate that PIFU can be an effective, efficient, and safe way to reduce the number of outpatient visits in patients with axSpA. The results from ReMonit suggest that remote monitoring might not always be required for safe PIFU, although it should be noted that this was a single-centre study with strict eligibility criteria, possibly limiting generalizability of results.

## PERSPECTIVE OF PATIENTS AND HEALTHCARE PROVIDERS ON PIFU AND REMOTE MONITORING

Qualitative studies on PIFU and remote monitoring in (ax)SpA have additionally provided insight into the experiences of patients and HCPs with this new care approach. Patients in the intervention arms of both the TeleSpA (PIFU/TM) and ReMonit (remote monitoring and PIFU) trials reported positive experiences in semi-structured interviews, highlighting appreciation for the ability of PIFU and remote monitoring to reduce unnecessary visits during flare-free periods, thereby reducing waiting and travel times [15^▪^,16^▪^]. Additionally, improvements in disease insight, patient empowerment, autonomy, and shared decision-making were expressed. These improvements were attributed to a greater sense of independence promoted by PIFU and access to personal health data through using remote monitoring tools. Altogether, HCP-patient relationships and communication were not negatively affected. A third related study, the TeleSpactive mixed methods study, further reinforced these benefits [17^▪^]. In this study, patients with stable axSpA used a mobile health application and C-reactive protein (CRP) self-testing for 6 months to monitor disease activity, with an optional in-person visit at 3 months if considered necessary through shared decision-making via telephone. Patients indicated widespread satisfaction with all components of this care approach. However, several concerns raised by patients should be acknowledged. Loss of a ‘safety net’ and emotional support due to reduced face-to-face contact with HCPs, as well as feelings of insecurity and potential exclusion of vulnerable populations arising from increased reliance on technology, were the primary disadvantages voiced [15^▪^,16^▪^]. Even so, predominantly positive experiences were reflected, with all TeleSpA participants indicating willingness to continue receiving care through PIFU/TM and recommend this approach to others [15^▪^]. Similarly, 96% of the ReMonit cohort reported willingness to use remote care in an ancillary survey [16^▪^,18^▪^].

Comparable experiences with PIFU and remote monitoring were expressed by HCPs in the TeleSpA trial. Illustratively, 12 of 13 (92%) surveyed HCPs were satisfied with the PIFU/TM intervention, and one was indifferent [15^▪^]. When interviewed, the vast majority considered PIFU/TM acceptable, safe, and able to facilitate good quality of care. While HCPs experienced little difference in terms of workload and time investment at the outpatient clinic, a noteworthy advantage was the increased flexibility in daily planning afforded by a reduced number of preplanned in-person appointments. This, in turn, contributed to improvements in workflow and allowed HCPs to allocate additional time to patients in greater need of consultation (e.g. those experiencing a flare). Several prerequisites for the implementation of PIFU/TM were nonetheless emphasized, including the integration of remote monitoring modalities with existing hospital systems to reduce administrative burden, and the availability of a supporting infrastructure that allows ad-hoc diagnostic testing and provides accessible logistical and technical support. Given the fulfilment of these conditions, all interviewed HCPs involved in the TeleSpA trial found providing care through PIFU/TM agreeable.

## CHALLENGES OF IMPLEMENTING PIFU AND REMOTE MONITORING IN PRACTICE

The merit of PIFU combined with remote monitoring is evident. However, in addition to the practical challenges with this approach encountered in trial settings, further recognized concerns warrant attention before implementation in practice. While remote monitoring can help mitigate some of these risks – including loss to follow-up, delayed care due to patient reluctance or inability to access services, and diagnostic delays when conditions are asymptomatic or patients fail to recognize the need for medical review – continuous vigilance is essential [19]. Particularly, remote monitoring of PROs, alongside laboratory tests (e.g. CRP, renal and liver function) conducted at the convenience of the patient in their own environment or via a home test, should be incorporated. This enables continuous assessment of disease activity through both subjective and objective measures, including recommended composite indices that require laboratory testing (e.g. ASDAS), and ensures adequate surveillance of medication safety [1]. It is also necessary to carefully select the right patient for PIFU and remote monitoring, and facilitate direct-access appointments through several means. Patients may further benefit from training and support on how to recognize, monitor, and self-manage their symptoms, as well as a hybrid approach combining PIFU with both remote monitoring and preplanned appointments once every (other) year, providing a ‘safety net’ and allowing for continued care planning [10,13].

The ideal patient for PIFU combined with remote monitoring is someone with stable disease (i.e. acceptable disease state and no changes in medication expected), who is digital and health literate, as well as motivated and compliant [15^▪^]. In patients that do not (initially) meet these prerequisites, PIFU could still be an option with additional support from HCPs. Patients with higher disease activity states, as measured by the ASDAS, may also be suitable, provided that both the patient and rheumatologist consider the disease state acceptable, with no changes in medication expected on the short term [12^▪▪^]. It is important to note that disease state in axSpA is largely assessed via PROs within the ASDAS, which may be influenced by concomitant diseases (e.g. osteoarthritis) not requiring treatment (changes), potentially resulting in an overestimation of disease activity scores [12^▪▪^]. Simultaneously, it should also be recognized that not every patient is a suitable candidate for PIFU and remote monitoring. If, despite extra guidance and consideration, disease-related or contextual prerequisites cannot be met, ‘traditional’ follow-up with preplanned visits should always remain an option [15^▪^].

A final consideration is the (very) low number of HCP-patient contacts that might result from PIFU and remote monitoring, which has several consequences. Firstly, medication tapering could become less frequent, eventually leading to overtreatment. It may therefore be advantageous for patients considering tapering to not participate in this follow-up approach. Secondly, lower contact frequencies could necessitate the use of mobile health applications for remote monitoring through electronic PRO measures. Employing these applications has the potential to enhance efficiency of care and aid decisions on whether to postpone a visit or conduct it remotely, especially for patients in an ID/LDA state [20]. Conversely, a serious threat when applying mobile health applications is poor adherence and high attrition rates [21]. Adherence is often achievable during the foreseeable timeframe of trial settings. For example, in the ReMonit trial, adherence was >80% over 18 months, albeit declining over time, with no specific patient characteristics significantly associated with adherence to PRO reporting [18^▪^]. In practice, however, sustained engagement with monitoring is required, and there is no guarantee that this behaviour will persist, as patients may eventually no longer find it useful or relevant to record their symptoms, particularly when their disease is inactive [22,23]. Consequently, patient education on the importance of remote monitoring when implementing PIFU is pivotal, and for patients that have been in an acceptable disease state for prolonged periods, the frequency and intensity of remote monitoring could possibly be minimized. Lastly, replacing frequent HCP-patient contacts with remote monitoring introduces logistical challenges, requiring a smoothly operating back office with a well-integrated digital infrastructure to manage large patient populations, as well as process electronically captured health data and (externally collected) laboratory results [15^▪^]. This may not be feasible in all healthcare settings, and could call for collaboration with an interprofessional team. Logistical feasibility is also linked to financial considerations, whereby the arrangement of adequate reimbursement for asynchronous remote follow-up (i.e. checking remotely-collected questionnaires and laboratory results) is necessary. As reimbursement pathways have been established for e-consultations in many countries, these could potentially also be used for this purpose. Altogether, these concerns represent important factors that still require careful consideration when adopting PIFU and remote monitoring.

## CONCLUSION

As an alternative to current practice, PIFU combined with remote monitoring results in significant and meaningful reductions in rheumatology visits, without significant changes in health outcomes (particularly disease activity). This approach also leads to healthcare cost savings. While implementation of PIFU and remote monitoring for axSpA in clinical practice holds potential, their inherent challenges must be addressed, for example through patient training and support on symptom recognition, monitoring, and self-management.

## Acknowledgements

*None.*

*This manuscript has been drafted, reviewed, and approved by all contributing authors.*

### Financial support and sponsorship

*None*

### Conflicts of interest

*M.L.S. has received honoraria from Novartis. C.W. has received honoraria from Novartis and research grants from AbbVie. A.v.T. has received honoraria from Novartis and Pfizer, research grants from AbbVie, Novartis, and UCB, and consulting fees from AbbVie, Johnson & Johnson, Novartis, and UCB.*

## REFERENCES AND RECOMMENDED READING

Papers of particular interest, published within the annual period of review, have been highlighted as:

▪ of special interest
▪▪ of outstanding interest
